# Use of Alendronate Sodium (Fosamax) to Ameliorate Osteoporosis in Renal Transplant Patients: A Case-Control Study

**DOI:** 10.1371/journal.pone.0048481

**Published:** 2012-11-20

**Authors:** Wen-Hung Huang, Shen-Yang Lee, Cheng-Hao Weng, Ping-Chin Lai

**Affiliations:** 1 Department of Nephrology, Chang Gung Memorial Hospital, Linkou, Taiwan, Republic of China; 2 Chang Gung University College of Medicine, Taoyuan, Taiwan, Republic of China; Baylor College of Medicine, United States of America

## Abstract

**Background:**

Renal transplant patients often have severe bone and mineral deficiencies. While the clinical effects of immunosuppressive agents like calcineurin inhibitors (CIs) and sirolimus on bone turnover are unclear, bisphosphonates are effective in bone recovery in these patients. Gender is significantly associated with osteoporosis and affects bone turnover, which is different in women and men. The effective gender-related site of action of bisphosphonates is unknown.

**Methods:**

Initially, we enrolled 84 kidney recipients who had received their transplants at least 5 months ago; of these, 8 were excluded and 76 were finally included in the study. First bone mineral density (BMD) at the lumbar spine, hip, and femoral neck was determined using dual-energy X-ray absorptiometry (DXA) between September 2008 and March 2009. These 76 patients underwent a repeat procedure after a mean period 14 months. Immunosuppressive agents, bisphosphonates, patients' characteristics, and biochemical factors were analyzed on the basis of the BMD determined using DXA.

**Results:**

After the 14-month period, the BMD of lumbar spine increased significantly (from 0.9 g/cm^2^ to 0.92 g/cm^2^, p<0.001), whereas that of the hip and femoral neck did not. Ordinal logistic regression analysis was used to show that Fosamax improved bone condition, as defined by WHO (p = 0.007). The use of immunosuppressive agents did not affect bone turnover (p>0.05). Moreover, in subgroup analysis, Fosamax increased the BMD at the lumbar spine and the hipbone in males (p = 0.028 and 0.03, respectively) but only at the lumbar spine in females (p = 0.022).

**Conclusion:**

After a long periods after renal transplantation, the detrimental effects of steroid and immunosuppressive agents on bone condition diminished. Short-term Fosamax administration effectively improves BMD in these patients. The efficacy of Fosamax differed between male and female renal transplant patients.

## Introduction

Patients maintained on dialysis for end-stage renal disease exhibit severe mineral and bone deficiencies. While renal transplantation restores defective kidney function in patients with chronic renal disease, the associated steroid and other immunosuppressive therapies continuously damage the bones [1], [2]; the expected correction of established bone lesions does not occur. Although transplantation can resolve many biochemical imbalances, such as hyperparathyroidism, associated with chronic renal failure, progressive loss of BMD in the trabecular bone often occurs early after renal transplantation [3]. Investigators have not agreed on the risk factors that are most strongly associated with reduced BMD [4], [5] after renal transplantation, except on an accumulated dose of steroid. At present, the use of biochemical markers of bone turnover in the serum or urine is not recommended for diagnosis [6]. The World Health Organization (WHO) defines osteoporosis as a condition in which the difference between the mean BMDs for the lumbar spine (LS), femoral neck (FN), or hip (H) of the patients and healthy young adults is more than 2.5 standard deviations (SDs), as measured by dual energy X-ray absorptiometry (DXA). Further, osteopenia is defined as a condition in which the difference between the mean BMDs of the patients and healthy young adults is between 1 and 2.5 SDs [6]. Several studies have shown the beneficial effects of bisphosphonates on post-transplantation osteoporosis [7]–[9]. Other studies have shown that calcineurin inhibitors (CIs) have deleterious effects on bone mineral metabolism in rats [10]–[12], and that at least one cyclosporine has a protective effect on bone [13]. Other immune-modifying drugs, such as azathioprine, mycophenolate mofetil, and sirolimus, which are used in conjunction with glucocorticoids and CIs, have not been shown to promote bone loss, neither experimentally nor clinically [14], [15]. Osteoporosis caused by portosystemic shunting [16], or by steroid or CIs through receptor activator of nuclear factor kappa-B ligand (RANKL)-dependent pathways, may be partially ameliorated using sirolimus [17]. Moreover, the physiology of bone turnover differs according to gender, particularly in menopausal women [18]–[21], and the efficacy of alendronate in the treatment of postmenopausal osteoporosis has been well established [22]. To our knowledge, the gender-related efficacy of alendronate in renal transplant subjects has rarely been reported. The aim of this randomized case-control study was to assess the impact of immunosuppressive agents and alendronate on BMD, as estimated by DXA, and to determine whether the response to alendronate in renal transplant subjects is gender-dependent.

## Materials and Methods

This case-control study complied with the guidelines of the Declaration of Helsinki and approved by the Medical Ethics Committee of Chang Gung Memorial Hospital, a tertiary referral center located in the northern part of Taiwan. Since this study involved retrospective review of existing data, the Institutional Review Board approval was obtained, but without specific informed consent from patients. In addition, all individual information was securely protected (by delinking identifying information from main data set) and available to investigators only. Furthermore, all the data were analyzed anonymously. On the other hand, if this study involved retrospective review of existing data plus retrospective analysis of remaining biological samples, both Institutional Review Board approval and specific informed consent must be obtained from all patients. The Institutional Review Board of Chang Gung Memorial Hospital has specifically waived the need for consent. Finally, all primary data were collected according to strengthening the reporting of observational studies in epidemiology guidelines. The form described above was referenced from the Liu et al.'s publication [23].

### Study population

We randomly enrolled 84 kidney recipients (40 men and 44 women) who had undergone transplantation at least 5 months ago. We used DXA to obtain BMD measurements of the lumbar spine (LS), left hip (H), and femoral neck (FN) between September 2008 and March 2009 [24]. Bone condition was defined on the basis of the WHO criteria: a BMD value >2.5 standard deviations (SD, T score) below the young adult mean indicated osteoporosis and that between 1.0 and 2.5 SDs below the mean indicated osteopenia. The immunosuppressive agents that the patients had received included prednisolone (5 mg/tablet), cyclosporine (25 mg/tablet and 100 mg/tablet), tacrolimus (0.5 mg/tablet and 1 mg/tablet), sirolimus (1 mg/tablet), and mycophenolate (250 mg/tablet). Fosamax (alendronate sodium; 70 mg/tablet, 70 mg per week) was administered to the patients who were initially diagnosed with osteoporosis. Fasting blood levels of serum creatinine (Cr), blood urea nitrogen (BUN), calcium, inorganic phosphate, and uric acid were obtained. The patients' medical records were studied for the history of diabetes mellitus (DM), smoking frequency, alcohol intake, and hepatitis B (HBV), hepatitis C (HCV), and cytomegalovirus (CMV) infections. All the doses of immunosuppressive agents administered between the 2 BMD measurements were considered as the accumulated dose. After 14±1.6 months of follow-up, the 76 remaining patients (8 of the 84 patients were excluded—2 subjects had died, 2 had graft failure, and the initial BMD measurements of 4 patients was lost) received a second measurement of BMD and fasting blood tests.

### Precautions and contraindications for the use of Fosamax

The first DXA report was obtained between September 2008 and March 2009. Fosamax (70 mg per week) was administered to the patients diagnosed with osteoporosis based on this DXA report unless 1 or more of the following conditions was present: bisphosphonate allergy; blood calcium levels <8 mg/dL; active stomach problems (e.g., esophagitis, gastritis, or ulcers); renal insufficiency (estimated glomerular filtration rate [eGFR] <30 mL/[min·1.73 m^2^] or serum Cr level >3 mg/dL); difficulty swallowing or the inability to stand/sit upright for at least 30 min, and pregnancy and breastfeeding. The patients did not receive Fosamax prior to obtainment of the first DXA data. Furthermore, the patients were informed that they should take the drug only upon rising for the day with 3 to 4 swallows of water and that they should stand, walk, or sit and fast for 30–45 min afterwards, and then eat breakfast. Lying down or reclining after taking the drug is prohibited. At least 30 min should pass after the intake of alendronate before taking supplements or other drugs.

### Immunosuppressive protocol

In our hospital, we mainly use a CI-based immunosuppressive regimen in the initial months of transplantation. Most of our patients also receive mycophenolic acid plus prednisolone during this stage. Immediately after transplantation, the targeted cyclosporine concentration at 2 h post-dose (C_2_) is approximately 1300–1100 ng/mL and the tacrolimus trough level is maintained at approximately 12–10 ng/mL. These concentrations are tapered gradually in the first year. In patients that have been transplanted for more than 12 months, the cyclosporine C_2_ level is maintained at approximately 500–600 ng/mL and tacrolimus level at 3–4 ng/mL. Prednisolone is maintained at 1.25–10 mg per day, according to patient's condition. An mTOR inhibitor is added to the regimen if the patient's condition is suitable (proteinuria <800 mg/day and eGFR >40 mL/[min·1.73 m^2^]). The trough level of the mTOR inhibitor is maintained at approximately 3–8 ng/mL. Once the mTOR inhibitor has been added, the CI and mycophenolic acid dose are cut by 50% overnight, while prednisolone is maintained at the same dosage. Subsequently, the CI dose is tapered as much as possible. Most of the patients in this study received only 25 mg cyclosporine or 0.5 mg tacrolimus per day if an mTOR inhibitor was used.

### Statistical analysis

The data, given as median and interquartile ranges in non-normal distribution variables, are expressed as mean ± SD in normal distribution variables. The paired *t* test and the Wilcoxon signed-rank test were used to compare data of the patients at presentation and follow-up. The Kruskal–Wallis test and one-way analysis of variance (ANOVA) were performed to compare data of the different bone conditions defined by the WHO criteria. Comparisons among groups were performed using the Mann–Whitney test and Student's *t* test. We used multivariate ordinal logistic regression to test the expected value between clinical variables and the change of bone condition (grade 1: change to better, grade 2: no change, and grade 3: deterioration; change to better: from osteoporosis to osteopenia or normal, or from osteopenia to normal; no change: no change in bone condition at start and follow-up; deterioration: from normal to osteopenia or osteoporosis, or from osteopenia to osteoporosis), as defined by WHO. The Chi-square test was used to determine the correlation between the 2 binary variables; a *p* value <0.05 was considered statistically significant. All statistical analyses were performed using the Statistical Package for the Social Sciences (SPSS) Version 12.0 for Windows (SPSS Inc., Chicago, IL, USA).

## Results

### Characteristics of the study population

After a follow-up period of 14±1.6 months, 76 subjects received a second BMD measurement. Among these patients, 12 had a medical history of DM; 10 were infected with HBV, 15 with HCV, and 13 with CMV; 10 men were habitual tobacco users, and 8 men and 1 woman regularly consumed alcohol. Thirty-four patients received Fosamax, 57, prednisolone; 55, mycohenolate; 30, tacrolimus; 26, cyclosporine; and 34, sirolimus. Eight patients (11%) received a single immunosuppressive agent, 21 patients (28%) received 2 immunosuppressive agents, 36 (47%) received 3, and 11 (14%) received 4.

### Bone mineral density (BMD) at baseline and at follow-up

Table 1 shows the changes in BMD and blood biochemistry after 14±1.6 months of follow-up. In the 76 patients, calcium level decreased from 9.45±0.51 mg/dL to 9.29±0.52 mg/dL (p<0.001) and albumin from 4.42±0.29 g/L to 4.34±0.4 g/L (p = 0.009), both levels were still within the normal range. However, BMD of the lumbar spine increased from 0.9 to 0.92 g/cm^2^, (p<0.001). No correlation between the use of Fosamax and that of the immunosuppressive agents could be demonstrated, as determined by the Chi-square test (p>0.05). No patient who received Fosamax exhibited a deterioration in the condition of his bone structure, as defined by WHO criteria. Thirty patients showed no change in their bone condition, but 4 showed improvement. In patients who had not received Fosamax, 6 (14%) showed deterioration and 1 (2%) showed improvement.

**Table 1:** Data reported at baseline and at follow-up, n = 76.

	Baseline	follow-up	P value
**LS-BMD (g/cm^2^)**	0.90±0.14	0.92±0.14	<0.001
**H-BMD (g/cm^2^)**	0.81±0.14	0.81±0.14	NS
**FN-BMD (g/cm^2^)**	0.68±0.12	0.69±0.13	NS
**LS T**	−1.53±1.24	−1.32±1.26	<0.001
**H T**	−1.76±0.97	−1.68±1.07	NS
**FN T**	−2.45±0.96	−2.42±1.02	NS
**Smoking**	10/76		
**Alcohol**	9/76		
**DM**	12/76		
**HBV**	10/76		
**HCV**	15/76		
**CMV**	13/76		
**BUN (mg/dL)**	19.7±10.0	19.8±10.9	NS
**Cr (mg/dL)**	1.18±0.54	1.20±0.54	NS
**Ca (mg/dL)**	9.45±0.51	9.29±0.52	<0.001
**P (mg/dL)**	3.21±0.54	3.23±0.59	NS
**Uric acid (mg/dL)**	6.24±1.68	6.45±1.70	NS
**Albumin (g/L)**	4.42±0.29	4.34±0.40	0.009
**TC (mg/dL)**	201±44	190±46	NS
**TG (mg/dL)**	147±82	148±113	NS
**Normal**	5/76	5/76	
**Osteopenia**	30/76	29/76	
**Osteoporosis**	41/76	42/76	

At follow-up, LS-BMD was significantly greater than its initial value. The albumin and calcium levels also had decreased significantly, but they remained within normal range.

Abbreviations: LS-BMD, lumbar spine bone mineral density; H-BMD, hip bone mineral density; FN-BMD, femoral neck bone mineral density; T, number of standard deviations (SD) above or below the mean value of a sex-matched, young adult mean of BMD. DM, diabetes mellitus; BUN, blood urea nitrogen; Cr, blood creatinine; Ca, serum calcium; P, serum inorganic phosphate; TC, serum total cholesterol; TG, serum triglyceride; HBV, hepatitis B virus infection; HCV, hepatitis C virus infection; CMV, cytomegalovirus infection; NS, no significance, p>0.05.

### BMD in patients with osteoporosis and without

**Figure 1:**
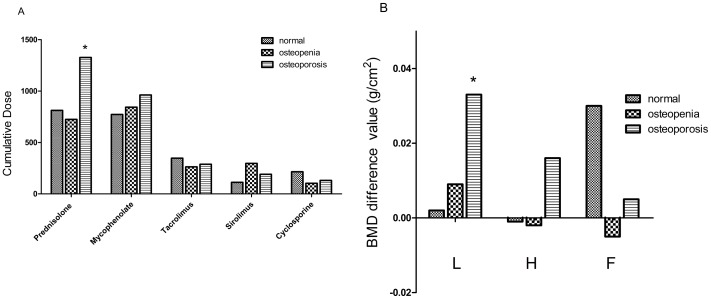
Cumulative dose of immunosuppressive agents and bone mineral density change between three bone conditions by WHO. The patients were divided, according to their baseline DXA, into normal (n = 5), osteopenia (n = 30), and osteoporosis (n = 41) groups. The osteoporosis group received a significantly greater cumulative prednisolone dose than did the osteopenia group (1326.5 mg vs. 724.5 mg; p = 0.005; Figure 1A), and the increase in lumbar spine bone mineral density was also significantly greater in the osteoporosis group (0.033 g/cm^2^ vs. 0.009 g/cm^2^; p = 0.028; Figure 1B). The drugs included in the analysis of cummulative immunosuppresive therapy were prednisolone, 5 mg; mycophenolate, 250 mg; tacrolimus, 0.5 mg; sirolimus, 1 mg; and cyclosporine, 100 mg. Abbreviations: L, lumbar spine; H, hipbone; F, femoral neck. *Statistical significance at p<0.05.

In 41 patients with and 35 without osteoporosis at baseline, the lumbar spine bone density increased (from 0.83 to 0.86 g/cm^2^ [p<0.001] and from 0.99 to 1.0 g/cm^2^ [p = 0.02]; respectively) after the mean 14-month follow-up period, but hip and femoral neck densities did not (Table 2). In order to detect any difference in BMD due to the use of immunosuppressive agents in different conditions of the bone, the patients were divided into 3 groups: normal, osteopenia, and osteoporosis, on the basis of the initial DXA findings. The osteoporosis group received a greater cumulative steroid dose than the osteopenia group (1326.5 mg vs. 724.5 mg; p = 0.005; Figure 1A), and the increase in the lumbar spine BMD was greater in the osteoporosis group (0.033 g/cm^2^ vs. 0.009 g/cm^2^; p = 0.028; Figure 1B). Otherwise, the cumulative dose of immunosuppressive agents among the 3 groups did not differ significantly (p>0.05; Figure 1A). Interestingly, of our 41 osteoporosis patients, 7 did not receive Fosamax due to their intolerance of the side effects. Among those 41 patients, those administered Fosamax showed a greater increase in BMD (0.035 g/cm^2^ vs. 0.003 g/cm^2^) but not significant (p>0.05).

**Table 2:** Comparison of patients with (41) and without (35) osteoporosis at presentation and follow-up.

	Non-osteoporosis patients (35)			Osteoporosis patients (41)			P value
	Baseline	1^st^ follow up	P value	Baseline	1^st^ follow up	P value	
**LS-BMD(g/cm^2^)**	0.99±0.12	1.00±0.13	0.02	0.83±0.11	0.86±0.12	<0.001	
**H-BMD(g/cm^2^)**	0.91±0.12	0.90±0.13	NS	0.72±0.11	0.73±0.10	NS	
**FN-BMD (g/cm^2^)**	0.78±0.09	0.78±0.11	NS	0.605±0.058	0.607±0.066	NS	
**LS T score**	−1.0 [−1.6,−0.2]	−0.9 [−1.5,0.3]	0.047	−2.15±1.02	−1.9±1.07	<0.001	
**H T score**	−1.06±0.65	−1.01±0.89	NS	−2.35±0.79	−2.25±0.86	NS	
**FN T score**	−1.59±0.68	−1.68±0.78	NS	−3.14±0.47	−3.09±0.54	NS	
**Gender (F/M)**		16/19			24/17		
**Smoking**		5/35			5/41		
**Alcohol**		5/35			4/41		
**DM**		7/35			5/41		
**BUN (mg/dL)**	17.7±7.6	17.7±6.3	NS	21.27±11.77	21.55±13.46	NS	NS*
**Cr (mg/dL)**	1.09±0.45	1.15±0.41	0.043	1.24±0.6	1.28±0.62	NS	NS*
**Ca (mg/dL)**	9.52±0.49	9.33±0.56	0.002	9.4±0.53	9.24±0.49	0.007	NS*
**P (mg/dL)**	3.22±0.58	3.22±0.57	NS	3.2±0.52	3.25±0.62	NS	NS*
**Uric acid (mg/dL)**	6.0±1.7	6.6±1.9	NS	6.44±1.73	6.26±1.59	NS	NS*
**Albumin (g/L)**	4.48±0.31	4.39±0.43	0.035	4.38±0.27	4.31±0.41	NS	NS*
**TC (mg/dL)**	198.1±40.2	190.5±44.3	NS	203.67±47.5	187.78±49.4	NS	NS*
**TG (mg/dL)**	159.9±85.5	152.5±106.3	NS	137.77±79.35	146.03±129.37	NS	NS*
**Normal**	5	5		0	0		
**Osteopenia**	30	25		0	4		
**Osteoporosis**	0	5		41	37		
***Cumulative dose of immunosuppressant agent***							
**Prednisolone (mg)**		872±730			1326.5±961		p = 0.003
**Mycophenolate (tablets)**		833.6±823.8			962.2±812.5		NS*
**Tacrolimus (tablets/1 mg)**		275.0±393.6			288.1±447.1		NS*
**Sirolimus (tablets)**		270.8±302.0			191.5±301.1		NS*
**Cyclosporine (100 mg tablets)**		119.20±210.85			131.12±177.79		NS*
***Increase in BMD***							
**LS-BMD (g/cm^2^)**		0.011±0.027			0.030±0.028		p = 0.005
**H-BMD (g/cm^2^)**		−0.005±0.03			0.007±0.036		NS*
**FN-BMD (g/cm^2^)**		0.003 ±0.052			0.002±0.031		NS*

The mean follow-up period was 14 months. In both the non-osteoporosis and the osteoporosis group, the LS-BMD significantly increased. At the end of the period, the cumulative dose of prednisolone and the LS-BMD differential were greater in the osteoporosis group.

NS*: p>0.05 between the osteoporosis and non-osteoporosis group.

Abbreviations: LS-BMD, lumbar spine bone mineral density; H-BMD, hip bone mineral density; FN-BMD, femoral neck bone mineral density; T score, number of standard deviations (SD) different from the mean value of the corresponding gender-matched young adult mean BMD. DM, diabetes mellitus; BUN, blood urea nitrogen; Cr, blood creatinine; Ca, serum calcium concentration; P, serum inorganic phosphate level; TC, serum total cholesterol level; TG, serum triglyceride level; NS: not significant, p>0.05.

### Factors associated with bone turnover

**Figure 2:**
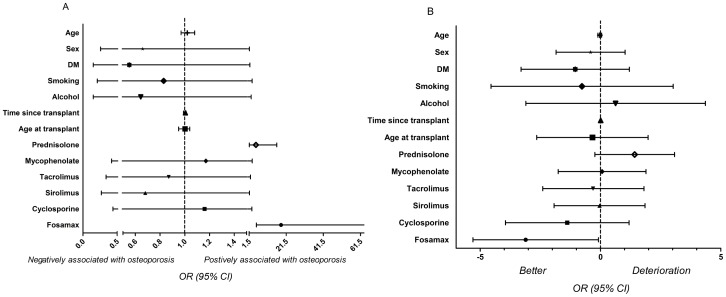
Clinical variables associated with bone condition change. A binary (non-osteoporosis and osteoporosis in follow-up) logistic regression analysis was performed to identify the variables associated with osteoporosis. The dependent variable was non-osteoporosis or osteoporosis. The independent variables were age, sex, DM, smoking, alcohol consumption, age at transplantation, time since transplant, use of immunosuppressive agents and use of Fosamax. Both the use of prednisolone (odds ratio [OR], 5.18; 95% confidence interval [CI], 1.6–16.4; p = 0.005) and the use of Fosamax (OR, 18.75; 95% CI, 5.42–64.76; p<0.001) were associated with the symptoms of osteoporosis (Figure 2A). In an ordinal logistic regression with multivariate analysis of the change of bone condition (grade 1, changed to the better; grade 2, no change; and grade 3, deterioration, as defined by WHO criteria and clinical variables), after adjusting for age, sex, status of diabetes (DM), smoking, alcohol consumption, time since transplant, age at transplant, and use of prednisolone, the use of Fosamax (OR, −3.115; 95% CI, −5.364 to −0.866; p = 0.007) was found to be associated with a positive prognosis (Figure 2B).

To deepen our investigation of the influence of clinical features on bone condition, we used a univariate binary logistic regression to evaluate the association between bone condition (osteoporotic and not osteoporotic at follow-up) and the clinical variables in 76 patients. The use of both prednisolone (odds ratio [OR], 5.18; 95% confidence interval [CI], 1.6–16.4; p = 0.005) and Fosamax (OR, 18.75; 95% CI, 5.42–64.76; p<0.001) showed an association in patients with osteoporosis (Figure 2A). In an ordinal logistic regression with multivariate analysis of the change in bone condition (grade 1, improvement; grade 2, no change; and grade 3, deterioration; as defined by WHO criteria) and the clinical variables, after adjusting for age, sex, status of diabetes, smoking, alcohol consumption, time since transplantation, age at transplant, and use of prednisolone, the use of Fosamax (OR, −3.115; 95% CI, −5.364; −0.866; p = 0.007) was found to be associated with a positive prognosis (Figure 2B).

### Gender differences in the effect of Fosamax on bones

In our study, we found no gender-related differences in bone turnover of renal transplant patients during the mean 14-month follow-up period. Seeking a gender-related difference in the physiology of bone turnover, we examined the bone response to Fosamax in the 2 sexes. We found no differences in bone turnover with respect to age, time since transplant, or the changes in blood values for creatinine, albumin, or calcium, and neither was the change in BMD or the cumulative immunosuppressive agents different in the 2 sexes. However, when we compared BMD before and after Fosamax treatment within the male and female groups, we found Fosamax to be more effective in men than in women. Among the patients who received Fosamax, BMD in the lumbar spine and the hip (p = 0.028 and p = 0.03, respectively) increased in 14 men; however, the increase in the BMD was observed only in the lumbar spine (p = 0.022; Table 3) in 20 women. Among the above-mentioned men and women who used Fosamax, the BMD difference values were not different (p>0.05). Thus, we find that the sites of action of Fosamax differ across the 2 sexes.

**Table 3:** Comparison of the use and non-use of Fosamax in men and women.

	Male patients (36)			Female patients (40)			P value
	Non-Fosamax (22)	Fosamax (14)	P value	Non-Fosamax (20)	Fosamax (20)	P value	
**Age (years)**	48±10.4	51.9±9.0	NS	49.7±7.6	53.3±8.8	NS	NS*
**Time since transplant (months)**	92±68.1	103.7±59.4	NS	61.4±39.6	97.6±72.9	NS	NS*
**Creatinine difference value (mg/dL)**	0 (−0.072, 0.122)	0.04 (−0.09, 0.21)	NS	0.05 (−0.035, 0.225)	0.01 (−0.075, 0.16)	NS	NS*
**Albumin difference value (g/L)**	−0.06 (−0.21, 0.08)	0.01 (−0.33, 0.18)	NS	−0.02 (−0.26, 0.047)	−0.1 (−0.21, 0.15)	NS	NS*
**Calcium difference value (mg/dL)**	−0.3 (−0.4, 0)	−0.2 (−0.4, 0.025)	NS	−0.15 (−0.27, 0)	−0.1 (−0.5, 0.17)	NS	NS*
***BMD difference value***							
**LS-BMD difference value (g/cm^2^)**	0.018 (−0.002, 0.036)	0.039 (0.02, 0.056)	0.028	0.004 (−0.004, 0.033)	0.032 (0.01, 0.051)	0.022	NS*
**H-BMD difference value (g/cm^2^)**	−0.0085 (−0.025, 0.015)	0.015 (−0.0035, 0.029)	0.03	0.003 (−0.015, 0.019)	0.023 (−0.043, 0.039)	NS	NS*
**FN-BMD difference value (g/cm^2^)**	0.004 (−0.021, 0.030)	0.0095 (−0.0065, 0.03)	NS	−0.0015 (−0.024, 0.017)	−0.0035 (−0.024, 0.016)	NS	NS*
***Cumulative dose of Immunosuppressive agents***							
**Prednisolone (mg)**	1054±700	1477±1072	NS	434 (0, 616)	1295 (751, 2151)	<0.001	NS*
**Mycophenolate (tablets)**	971±933	1079±902	NS	613±487	994±856	NS	NS*
**Tacrolimus (tablets/1 mg)**	0 (0, 178)	0 (0, 677)	NS	0 (0,705)	147 (0, 770)	NS	NS*
**Sirolimus (tablets)**	350 (0, 556)	0 (0, 425)	NS	0 (0, 383)	0 (0, 385)	NS	NS*
**Cyclosporine (tablets/100 mg)**	0 (0,402)	0 (0, 404)	NS	0 (0, 192)	0 (0, 253)	NS	NS*

Under similar conditions (characteristics, BMD differences, and cumulative use of immunosuppressive agents) the use of Fosamax in male patients increased the BMD of both lumbar spine and hip bone, but in female patients it increased the BMD only of lumbar spine.

NS*: p>0.05 between male and female patients.

Abbreviations: LS-BMD, lumbar spine bone mineral density; H-BMD, hip bone mineral density; FN-BMD, femoral neck bone mineral density;

NS, no significance, p>0.05.

## Discussion

In this study, we have shown that short-term weekly use of Fosamax can improve both BMD and bone condition, in accordance with the WHO criteria, regardless of effect of immunosuppressive agents after a long period after renal transplantation. In renal transplant subjects with osteoporosis, Fosamax improved the BMD of the lumbar spine. Although the bone condition after renal transplantation did not vary according to gender, the bone regions in which Fosamax was effective did vary.

An increase in bone mass loss is multifactorial and is affected by age [21], sex [19]–[21], renal function, and duration of time for which the patient was on dialysis before transplantation [20]. A major influencing and well-known factor causing increased loss of bone mass is high-dose steroid therapy during the early period after transplantation and continuous long-term steroid administration [2]. CIs such as cyclosporine and tacrolimus also are known to have serious effects and cause rapid and severe bone losses in both animal models and humans. The role of T-lymphocyte action via RANKL seems to be of essence in triggering bone loss [14]. Other immune-modifying drugs such as azathioprine, mycophenolate mofetil, and sirolimus, which are used in conjunction with glucocorticoids and CIs, have—neither experimentally nor clinically—been shown to promote bone loss. Recent studies [17], [25] suggest that sirolimus could promote an osteoclastic balance between the effects of steroid and of calcineurine inhibitors. Moreover, under sirolimus-based maintenance immunosuppression [26] after bone surgery, no radiologic advantage or disadvantage to bone healing was noted. Therefore, in our clinical estimation, a bias may exist between the biochemical markers and DXA–WHO criteria in osteoporosis to evaluate bone condition.

Interestingly, the lumbar spine BMD in our 35 non-osteoporotic patients increased slightly. As was true with the steroids, the cumulative dose of immunosuppressive agents in the non-osteoporosis and osteoporosis groups did not differ. In the past decade, the use of corticosteroids in the peritransplantation period has been dramatically reduced and replaced by CIs and other adjunctive agents. To our knowledge, the first 3 to 6 months after transplantation is the critical period for the loss in bone mass [4], [27], [28]. In a long-term study, the ongoing accelerated lumbar bone mass loss was 1.7±2.8% per year [29], but in the 12 months following cardiac transplantation, the LS BMD value was restored to that at the time of transplant [30]. Twenty-four months after transplantation, the yearly loss of absolute BMD was parallel to the age-dependent physiological decline in absolute BMD [5]. In our study, the time since transplant of non-osteoporotic subjects was 78±60 months. From the studies cited above and from our observations during the long post-transplantation period, we can explain the slightly increased BMD of lumbar spine in our patients who did not receive Fosamax. Otherwise, as the use of immunosuppressive agents changes in the long period of renal transplantation, the effective power of steroid on bone turnover may be increasingly minor.

In this study, when we analyzed the subgroups of the subjects with osteoporosis, we observed that Fosamax was not equally effective in men and women in the different bone regions. Several previous studies have shown that bisphosphonates continued to improved bone mineral density after renal transplantation [7]–[9], [31], [32] and most of them showed the effect on both lumbar spine and femoral neck BMD. In a meta-analysis review of 1209 patients [33], treatment with bisphosphonates increased BMD in lumbar spine and femoral neck. No measurable change in the BMD of the hip area was observed. In another population-based longitudinal study [18], both the bone turnover and the sites of bone loss differed according to gender. In a 2-year double-blind trial of men with osteoporosis (mean age, 63 years), alendronate significantly increased bone mass of spine and hipbone and helped prevent vertebral fractures [34]. Iwamoto et al. [35] have suggested that although alendronate treatment in men effectively increased lumbar BMD from baseline, its efficacy appeared to be no greater than that in postmenopausal women with osteoporosis. Studies on the comparative effects in a renal transplant population of bisphosphonates according to gender and bone site are limited. In our study on men and women who presented no significant differences in the increase in their BMD with Fosamax, the 14 osteoporotic men showed improved BMD at the hip and lumbar spine and the 20 osteoporotic females responded well only at the lumbar spine.

Fosamax is absorbed and partitioned rapidly, with approximately 50% binding to the exposed bone surface and the remainder being excreted unchanged by the kidneys [36]. Therefore, Fosamax should be used carefully in patients with renal insufficiency or in anuric patients because of concerns regarding drug accumulation. The major side effect of Fosamax is ulceration of the esophagus, which may require hospitalization and intensive treatment. Gastric and duodenal ulceration may also occur [37], [38]. The co-administration of Fosamax and calcium, antacids, or oral medications containing multivalent cations interferes with the absorption of alendronate [38]. The short-term use of a low dose of Fosamax (40 mg per week) was safe in hemodialysis patients [39]. Alendronate treatment safely and effectively increased BMD and decreased fractures in women with normal to severely impaired renal function [40], and no differences in adverse events were observed in these women according to their renal function. The use of Fosamax for a mean of 14 months did not deteriorate renal function in male and female renal transplant patients (Table 3). To our knowledge, studies on the interaction between Fosamax and immunosuppressant agents are limited. The studies cited above and our own observations suggest that the interaction between these agents is subtle.

Although the limitations of our study with regard to its retrospective design and small sample size are evident, to our knowledge, these are the first data reporting the differences between men and women with regard to the sites at which Fosamax is effective in renal transplant patients; these findings refer to short-term treatment after a long post-renal transplantation period.

## Conclusions

In conclusion, this randomized case-control study has shown that short-term use of Fosamax increased BMD and that the effect of concomitant steroid was not significantly correlated with bone turnover. Moreover, Fosamax increased the BMD of the lumbar spine and hip significantly in men, but only in the lumbar spine in women. Immunosuppressive agents such as CIs, sirolimus, and mycohenolate were not correlated with any change in BMD.
